# Using novel deep learning models for rapid and efficient assistance in monkeypox screening from skin images

**DOI:** 10.3389/fmed.2024.1443812

**Published:** 2024-09-05

**Authors:** Jie Deng, Jingjie Liu, Chui Kong, Boyang Zang, Yue Hu, Meiyin Zou

**Affiliations:** ^1^School of Medical College, Jiangsu University, Zhenjiang, China; ^2^Department of Cardiology, First Affiliated Hospital of Dalian Medical University, Dalian, China; ^3^School of Information Science and Technology, Fudan University, Shanghai, China; ^4^School of Clinical Medicine, Tsinghua University, Beijing, China; ^5^Guang'anmen Hospital, China Academy of Chinese Medical Sciences, Beijing, China; ^6^Department of Infectious Diseases, Affiliated Nantong Hospital 3 of Nantong University, Nantong, Jiangsu, China; ^7^Department of Infectious Diseases, Nantong Third People’s Hospital, Nantong, Jiangsu, China

**Keywords:** monkeypox, deep learning, self-attention mechanisms, auxiliary diagnostic, skin images

## Abstract

Monkeypox, a communicable disease instigated by the monkeypox virus, transmits through direct contact with infectious skin lesions or mucosal blisters, posing severe complications such as pneumonia, encephalitis, and even fatality. Traditional clinical diagnostics, heavily reliant on the discerning judgment of clinical experts, are both time-consuming and labor-intensive, with inherent infection risks, underscoring the critical need for automated, efficient auxiliary diagnostic models. In response, we have developed a deep learning classification model augmented by self-attention mechanisms and feature pyramid integration, employing attentional strategies to amalgamate image features across varying scales and assimilating *a priori* knowledge from the VGG model to selectively capture salient features. Aiming to enhance task performance and model generalizability, we incorporated different components into the baseline model in a series of ablation studies, revealing the contribution of each component to overall model efficacy. In comparison with state-of-the-art deep learning models, our proposed model achieved the highest accuracy and precision, marking a 6% improvement over the second-best model. The results from ablation experiments corroborate the effectiveness of individual module components in enhancing model performance. Our method for diagnosing monkeypox demonstrates improved diagnostic precision and extends the reach of medical services in resource-constrained settings.

## 1 Introduction

Since May 2022, outbreaks of monkeypox have successively emerged in multiple countries worldwide, currently affecting 112 countries with over 90,000 reported cases (1). Monkeypox (MPX) is a zoonotic disease caused by the monkeypox virus. This virus is highly infectious and can be transmitted to humans through contact with the bodily fluids, blood, secretions, and broken skin or mucous membranes of infected animals (2). Healthcare workers involved in the prehospital care, transportation, and disinfection processes face a relatively high risk of transmission when treating infectious diseases. The primary clinical manifestations of monkeypox infection include sudden onset, fever, and malaise, accompanied by a rash, which can be easily misdiagnosed as smallpox or mild chickenpox (3). This overlap in clinical presentation poses a significant challenge for early diagnosis of monkeypox virus infection, particularly in resource-limited settings where access to advanced laboratory diagnostic tools may be restricted. Therefore, early diagnosis of monkeypox virus infection presents a significant challenge (4).

Current diagnostic methods, including nucleic acid testing (PCR) (5), antibody and antigen detection, next-generation sequencing (NGS) (6), viral isolation and culture, and electron microscopy, are highly effective but require specialized equipment and trained personnel. The gold standard for laboratory testing of monkeypox is nucleic acid testing. The optimal diagnostic specimens should be collected from skin lesions, or the top of liquid contents of vesicles and pustules, as well as dry scabs. These methods are not always feasible in low-resource environments where monkeypox outbreaks are more likely to occur. Real-time PCR, known for its high accuracy and sensitivity, is the preferred laboratory testing method recommended by the World Health Organization. However, in regions with limited resources lacking PCR, computer-aided diagnostics of monkeypox lesions presents a promising alternative. It offers the potential for rapid screening and diagnosis without the need for extensive laboratory infrastructure, facilitating the early and rapid screening of suspected cases and aiding healthcare workers in tracing and curtailing the spread of the monkeypox virus. In recent years, with the swift advancement of machine learning, significant achievements have been made in medical imaging. Medical images often present unique challenges due to their intricate features, specific color channels, and the need for precise interpretation in a clinical context. These complexities have driven the adoption of deep learning techniques in medical imaging, supported by advancements in hardware and software. Sitaula and Shahi (7) achieved favorable results using deep learning and transfer learning techniques for the automatic detection of monkeypox skin lesions. Following this, Jaradat et al. (8) proposed an improved convolutional neural network (CNN) model to assist in the early detection and classification of human skin lesions (9), utilizing advanced transfer learning (TL) algorithms and ensemble methods. This innovation represents a novel approach for the early detection of monkeypox. Haque et al. presented an ensemble of fine-tuned deep learning models for monkeypox detection, showcasing a comparative study that highlights the effectiveness of such models in accurately identifying monkeypox cases (10). Additionally, a CNN-LSTM-based hybrid deep learning approach has been explored for sentiment analysis on monkeypox tweets, which underscores the versatility of deep learning methods in different applications related to monkeypox (11).

Despite the advancements in machine learning and medical imaging, existing deep learning models used for monkeypox detection face several limitations. These models often suffer from weak interpretability, which limits their clinical applicability, and insufficient feature extraction capabilities, which can reduce diagnostic accuracy (12, 13). There is a clear gap in developing deep learning models that not only achieve high accuracy but also provide meaningful insights into the diagnostic process.” “To address these challenges, our study proposes a novel approach by integrating an Attention mechanism module and a multi-feature pyramid into the deep learning model. The Attention mechanism allows the model to focus on specific parts of the input data, enhancing interpretability and accuracy. Additionally, the multi-feature pyramid attends to feature dimensions at different scales, improving the model’s ability to capture intricate details of monkeypox lesions. Furthermore, model distillation from a classic VGG model to a streamlined lightweight model aims to reduce the model size and enhance inference speed, making it more suitable for deployment in resource-constrained settings. Additionally, a multi-feature pyramid is introduced to attend to feature dimensions at different scales (14, 15). Ultimately, model distillation is implemented, transferring knowledge from a classic VGG model to our streamlined lightweight model (16), aiming to reduce the model size and enhance inference speed. Ablation experiments on different model components were conducted, and comparisons with various types of image classification networks were made. The results indicate that the improved model adequately recognizes the pathological features of monkeypox, exhibiting high accuracy. Lastly, the model’s interpretability was enhanced through visualization of its attention distribution.

The remainder of this paper is organized as follows. In Section 2, we describe the data collection and preprocessing methods used to prepare the Monkeypox Skin Lesion Dataset (MSLD) for training and testing. Section 3 details the construction of the proposed deep learning model, including the integration of the Attention mechanism module and multi-feature pyramid, as well as the model distillation process. In Section 4, we present the experimental setup, including the ablation studies and comparative analysis with other state-of-the-art models. Section 5 discusses the results, highlighting the performance metrics, confusion matrices, and ROC curves. Finally, Section 6 concludes the paper with a discussion on the implications of our findings, potential limitations, and future research directions.

The innovative aspects of this study are as follows:

1. We utilized a network with four residual connections as the baseline and further enhanced the algorithm for monkeypox pathology classification through the integration of a multi-feature pyramid, attention mechanism, and model distillation based on VGG. The aim was to achieve a lightweight and more precise identification of monkeypox, contributing to healthcare in economically underdeveloped regions.
2. Comprehensive ablation experiments were conducted, sequentially incorporating each model component into the baseline to assess their actual performance improvements. The findings indicate that each model component significantly enhances model performance.
3. Beyond ablation experiments, comparative studies were performed against several classic network architectures, such as Resnet50, EfficientNet, and DenseNet. The comparison results, visualized for clarity, demonstrated that our model achieves optimal performance.

## 2 Method

### 2.1 Data collection

**Figure 1:**
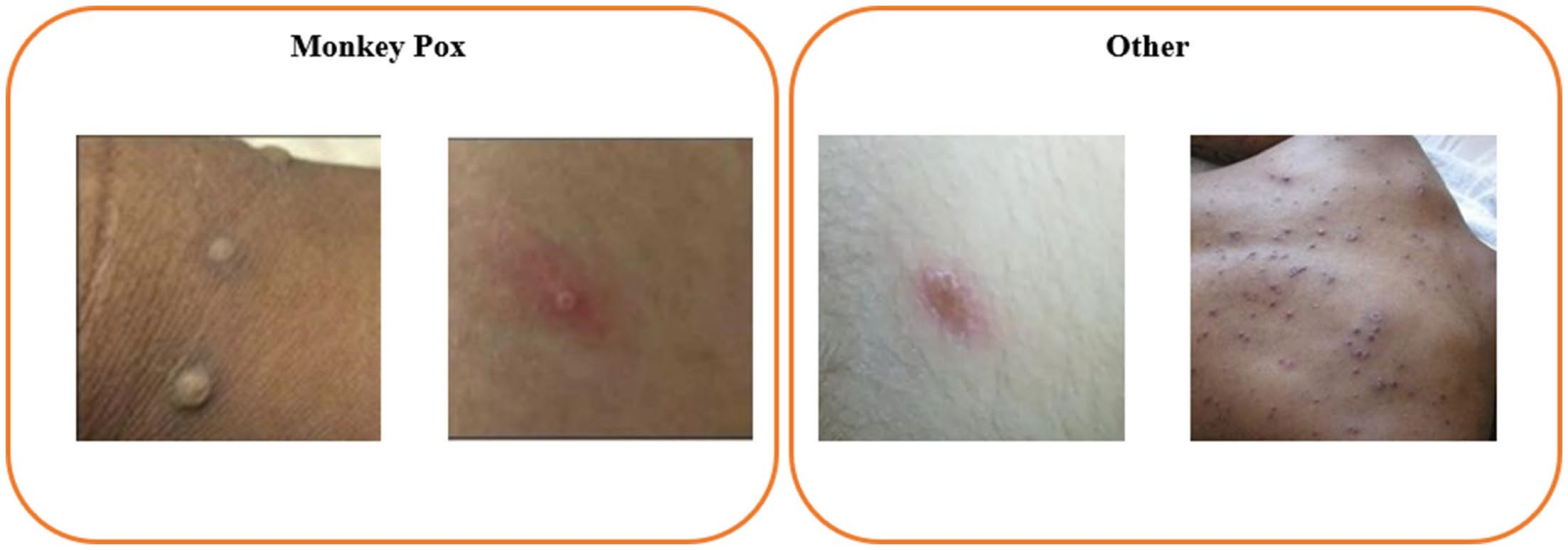
Example image from the Monkeypox Skin Lesion Dataset (MSLD).

The dataset utilized in this study is the publicly accessible Monkeypox Skin Lesion Dataset (MSLD) (17, 18), which was compiled by integrating images from a variety of sources, including news portals, websites, and publicly available case reports. The dataset comprises 228 images, categorized into two groups: 102 images of monkeypox and 126 images of other types. An example of the dataset is presented in Figure 1. Additionally, the MSLD offers results from preliminary data augmentation, expanding the image count to 1,428 for monkeypox and 1,764 for other categories, thus providing support for a more comprehensive and robust analysis.

### 2.2 Data preprocessing

In our research, the image-processed Monkeypox Skin Lesion Dataset (MSLD), comprising 3,192 images, was utilized. To enhance data quality and diversity, a series of preprocessing steps were applied during the data cleansing phase to the original dataset, including reflection, cropping, hue, saturation, contrast, and brightness jitter adjustments. These jitter adjustments simulate the variations in image appearance due to changes in lighting and environmental conditions, thereby improving the model’s adaptability and generalization.

To ensure the consistency of input data, we standardized the resolution of all images to 224 × 224 pixels, a common input size for convolutional neural networks, enhancing stability when processing images from varied sources. Additionally, we performed data augmentation techniques such as random horizontal flips and rotations, fortifying the model’s robustness against rotational variations that might be encountered in practical applications. Quantitative analysis was further conducted on the augmented images to ensure that the enhancement techniques did not introduce excessive noise or result in the loss of crucial features. The augmented set of images provided a more substantial and varied source of data for model training, covering a broader range of case variations, which significantly bolstered the model’s generalization capacity. This ensures that the model remains efficient and accurate in identifying monkeypox skin lesions amidst the diversity and variability of real-world scenarios.

### 2.3 Model construction

#### 2.3.1 Proposed deep learning-based model

In the medical field, classification models, particularly those based on deep learning, are revolutionizing the approach to diagnosis and disease identification. These models, by learning intricate patterns from medical images, can swiftly indicate the presence of diseases, at times matching or even surpassing the diagnostic acuity of human experts. For instance, convolutional neural networks (CNNs) (13), such as ResNet18 (12), and architectures like Vision Transformers (ViT) (19), have been successfully deployed for detecting and classifying conditions like skin cancer, diabetic retinopathy, and breast cancer screening. Their strength lies in the ability to process and analyze voluminous datasets, learning to discern complex, disease-characteristic patterns that are indicative of various pathologies. However, the limitations of these models are also quite evident. They may perform well on one dataset but exhibit diminished performance when generalized to new, different datasets, often due to insufficient feature extraction. To address this, we propose a novel monkeypox classification model aimed at bolstering the generalizability of deep learning models and enabling multi-scale feature extraction. This approach is designed to enhance model robustness and accuracy across diverse clinical imaging datasets.

**Figure 2:**
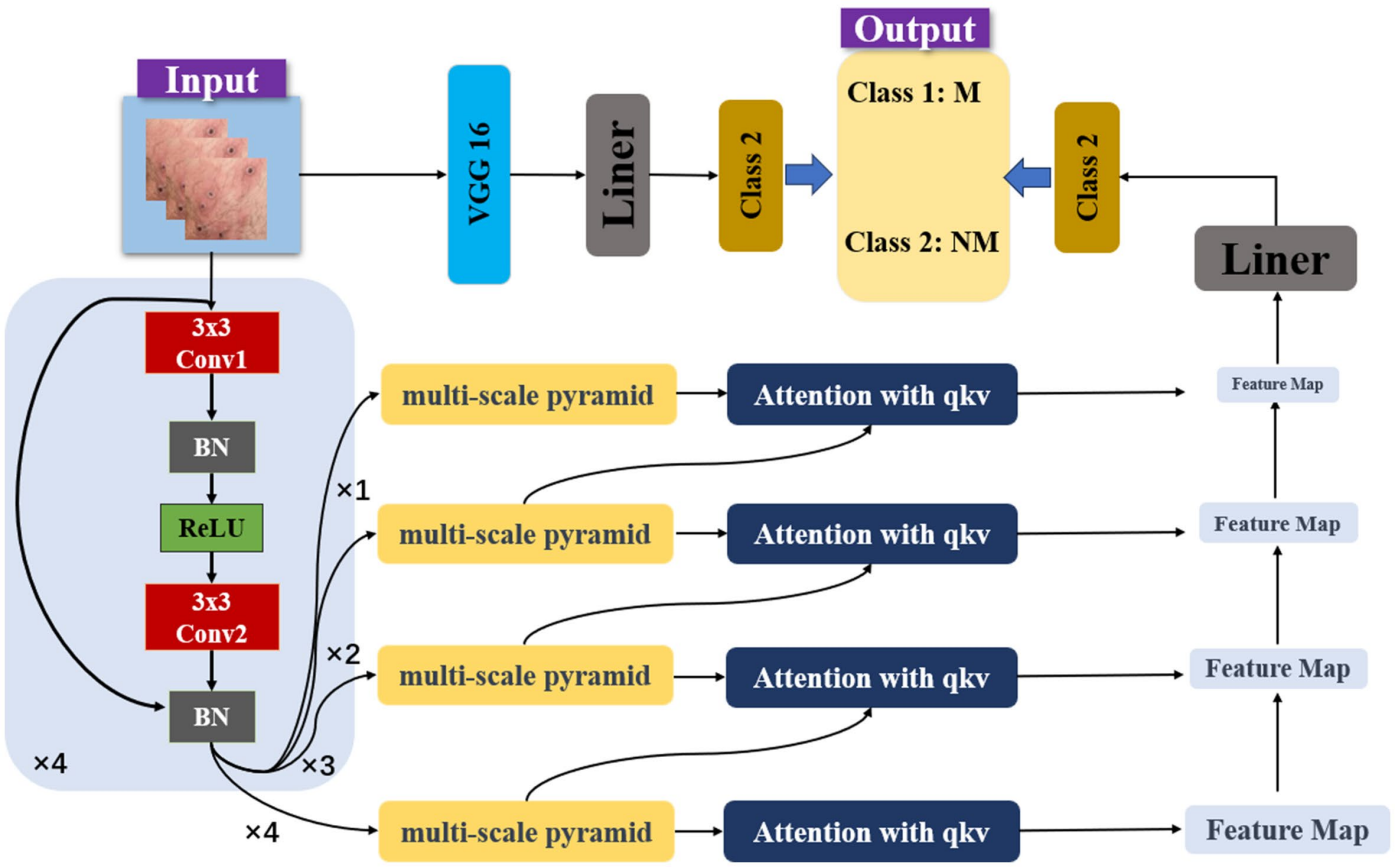
Model architecture.

As illustrated in Figure 2, our model is a sophisticated architecture designed for the precise classification of monkeypox skin lesion images. The input images are initially processed through 3 × 3 convolutional layers with Batch Normalization (BN) and ReLU activation functions, starting with 64 filters and increasing to 128, 256, and 512 filters in subsequent layers. These convolutional layers are replicated across subsequent layers, each responsible for capturing more abstract representations of the input data. At the core of our architecture is the multi-scale pyramid, enabling simultaneous processing of the image at various scales. This pyramid structure is pivotal for capturing features of different sizes and aspects, which is crucial for accurately identifying skin lesions with potential significant visual differences. Each level of the pyramid is further enhanced by an attention mechanism utilizing query (q), key (k), and value (v) vectors to selectively focus on the most informative parts of the feature map. An attention mechanism with 512-dimensional query (q), key (k), and value (v) vectors directs the model’s focus to critical regions. In the classification stage, the processed features from the pyramid and attention layers converge to two distinct output nodes, corresponding to the categories of monkeypox (Class 1: M) and non-monkeypox (Class 2: NM) lesions. A linear layer (Liner) is employed to map the high-dimensional feature data into the space of these two categories. Additionally, a learnable model fusion strategy is adopted, integrating with the classic VGG image classification network, adaptively adjusting the feature selection ratio.

The computational complexity of the model arises from both the convolutional layers and the attention mechanism integrated into the multi-scale pyramid structure. The convolutional layers contribute a complexity $O\left(N\times K^{2}\times C\times H\times W\right)$ , where N is the number of filters, K is the kernel size, C is the number of input channels, and H and W are the spatial dimensions of the feature maps. Each layer in the pyramid structure adds to this complexity, with varying dimensions and filter numbers at each scale. The attention mechanism introduces additional complexity with $O\left(H\times W\times d_{q}\times d_{k}\right)$ , where d_q and d_k represent the dimensions of the query and key vectors, respectively. As this mechanism operates across multiple scales, its complexity is cumulative. Overall, the model’s total complexity can be summarized as $O\left(\sum_{l=1}^{L}\left(N_{l}*K_{l}^{2}*C_{l}*H_{l}*W_{l}\right)+\sum_{s=1}^{S}\left(H_{s}*W_{s}*d_{q}*d_{k}\right)\right)$ .

In this study, a Feature Pyramid Network (FPN) (20) is employed to offer flexibility in adapting to skin lesions of varying sizes and shapes. The integration of attention mechanisms not only in each step of feature extraction but also across different feature hierarchy levels allows for inter-layer distribution of attentional tasks. In deep neural networks, feature representations at different levels signify different abstract concepts. Introducing attention mechanisms for cross-layer task allocation enables the model to capture interrelationships between different levels more effectively. The integration of VGG-16 allows the model to inherit and learn advanced visual feature representations from a classical pretrained network, thereby enhancing its accuracy and reliability in practical applications.

#### 2.3.2 Comparative model

This study employed commonly used models in the field of medical image classification and conducted comparisons with EfficientNet, MobileNet, CNN, RESNet50, RESNet18, and DenseNet (21, 22), including our model. Here, we introduce the relevant comparative models:

EfficientNet is known for its scaling strategy that balances depth, width, and resolution. In the field of medical imaging, EfficientNetB1 is widely used for tasks such as histopathological image analysis due to its efficiency and accuracy. MobileNet, designed for mobile and edge devices, prioritizes speed and efficiency. Its lightweight deep neural network architecture enables real-time analysis in medical imaging applications on mobile devices, supporting rapid diagnostics. CNN, the core of image analysis in deep learning, automatically learns the hierarchical structure of spatial features through convolutional layers. In medical imaging, generic CNNs are extensively applied to a variety of tasks, from lesion classification to tumor detection. ResNet18 and ResNet50, networks under the ResNet architecture, both utilize residual connections. ResNet50 is renowned for its deep residual learning framework and is used in the medical imaging field for lesion detection in X-ray and MRI scans, while ResNet18 is suitable for less complex datasets or situations requiring higher model interpretability. DenseNet is famed for its dense connection pattern, which helps to maximally transfer information flow, aiding in the detection of minute anomalies in images.

### 2.4 Ablation experiment

During the ablation study, we utilized a ResNet architecture with four layers of residual connections as the baseline model to ascertain the effectiveness of various model components. The experiment was conducted in three phases: initially, in the first phase, we incrementally introduced FPN (Feature Pyramid Network) layers to evaluate the performance of multi-scale feature maps. Subsequently, in the second phase, building on the FPN layer structure, we integrated an Attention module to investigate its potential in enhancing model precision. In the final phase, we merged the classical VGG model and selected weight parameters through simple linear layers, aiming to optimize the feature distribution. The ultimate goal of the ablation study was to validate the effectiveness of each component upon integration into the model.

### 2.5 Experiment setup

In this study, the dataset comprises 228 images, including 102 images of monkeypox and 126 of other categories. The data was partitioned in a 7:1:2 ratio for training, validation, and testing, respectively, allocating approximately 70% for training, 10% for validation, and 20% for testing. Data augmentation and preprocessing techniques increased the monkeypox category to 1,428 images and other categories to 1764 images, thereby enhancing the dataset’s diversity and the robustness of model training. The experimental setup utilized an NVIDIA GeForce RTX 3060 GPU and an Intel Core i5-12400F CPU, with the training environment based on Python 3.8. The learning rate was reduced by 70% every 10 epochs. All models were iterated until stable performance was achieved without overfitting, ensuring the reliability and effectiveness of the training process.

### 2.6 Model evaluation

In our research, we evaluated model performance using metrics such as accuracy, Matthews Correlation Coefficient (MCC) (23, 24), precision, and recall. Accuracy reflects the proportion of correctly identified observations across all predictions. The MCC, which can vary from −1 to 1, serves as a measure of binary classification effectiveness, with 1 denoting perfect accuracy, 0 a random guess level, and −1 complete disagreement between prediction and actual outcome. Precision quantifies the fraction of true positives among all positive predictions, whereas recall indicates the fraction of true positives out of the total actual positives. The equations for these metrics are detailed as follows:

## 3 Results

### 3.1 Our model result

In our study, we employed a Stochastic Gradient Descent (SGD) optimizer with a momentum of 0.9 and an initial learning rate of 0.001 for model training. The batch size was set to 32, coupled with a step learning rate scheduler that reduces the learning rate by 30% every 10 epochs. For model training, we utilized both cross-entropy and knowledge distillation loss functions. The model was iteratively trained over multiple epochs, learning from the training dataset and evaluated on the validation set to monitor performance metrics such as loss and accuracy. As training progressed (25), we generally observed a decrease in loss and an increase in accuracy until the model converged.

In our research focusing on the classification of monkeypox images, the newly proposed model demonstrated outstanding performance across various performance metrics. The model achieved an F1 score of 0.9834, indicating an exceptionally high balance between precision and recall, with the precision itself also reaching 0.9834, which signifies the model’s high accuracy in correctly identifying positive cases of monkeypox. The Matthews correlation coefficient (MCC) was 0.9617, reflecting a strong and reliable correlation between the predicted outcomes and the actual data. Additionally, the accuracy of the model was recorded at 0.9812, meaning that the vast majority of the classifications were correct. These metrics collectively suggest that the proposed model excels in classifying monkeypox images, demonstrating its potential as an effective tool in the diagnosis of the disease.

**Figure 3:**
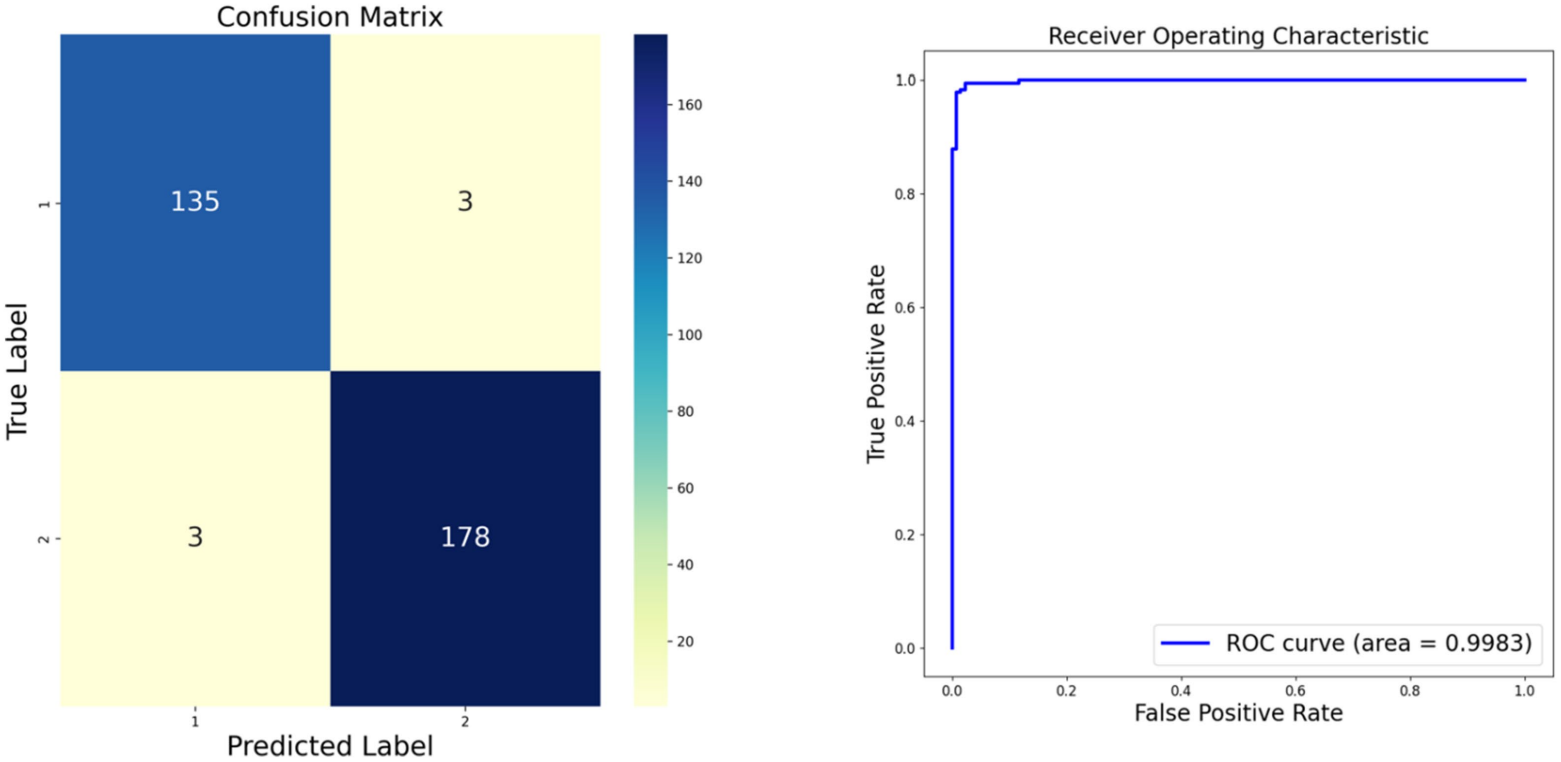
Our model confusion matrix and Receiver Operating Characteristic (ROC) curve.

As depicted in Figure 3, we present the confusion matrix and Receiver Operating Characteristic (ROC) curve for our model. The confusion matrix illustrates a high number of true positives and true negatives with minimal misclassifications, indicating strong diagnostic accuracy. Concurrently, the ROC curve demonstrates an excellent area under the curve (AUC) of 0.9983, further confirming the model’s outstanding discriminative ability between classes. Compared to existing literature, our model demonstrates superior accuracy (11). These results underscore the robustness and efficacy of our model in classifying monkeypox images.

### 3.2 Comparative experiment result

In our study, we compared the proposed model against several popular models, as depicted in Table 1, which includes efficientnetb1, mobilenetv2, a conventional cnn, RESNET50, RESNET18, and Desnet. The comparative results indicate that our model excels across all four key performance metrics. Specifically, our model attained an F1 score of 0.9834 and a precision of 0.9834, with a Matthews correlation coefficient (MCC) of 0.9617 and an accuracy of 0.9812. Compared to other models, for instance, efficientnetb1 which scored 0.9162 on F1 and 0.9266 on precision, our model surpasses the 0.98 mark in both metrics, demonstrating a clear advantage. Moreover, our model also shows a significant improvement in MCC and accuracy, particularly when contrasted with the standard cnn model, which only records an MCC of 0.6399 and an accuracy of 0.8119, further highlighting the superiority of our approach. Overall, our model outperforms the comparative models in all performance metrics, affirming its effectiveness and efficiency in the classification of monkeypox images.

**Table 1:** Comparative results of different models from the experiment.

	F1-score	Precision	MCC	Accuracy
efficientnetb1	0.9162	0.9266	0.8094	0.906
mobilenetv2	0.9628	0.9282	0.9133	0.9561
cnn	0.8182	0.906	0.6399	0.8119
RESNET50	0.97	0.957	0.9299	0.9655
RESNET18	0.9611	0.9665	0.9108	0.9561
Desnet	0.9665	0.9774	0.9239	0.9624
Our	0.9834	0.9834	0.9617	0.9812

**Figure 4:**
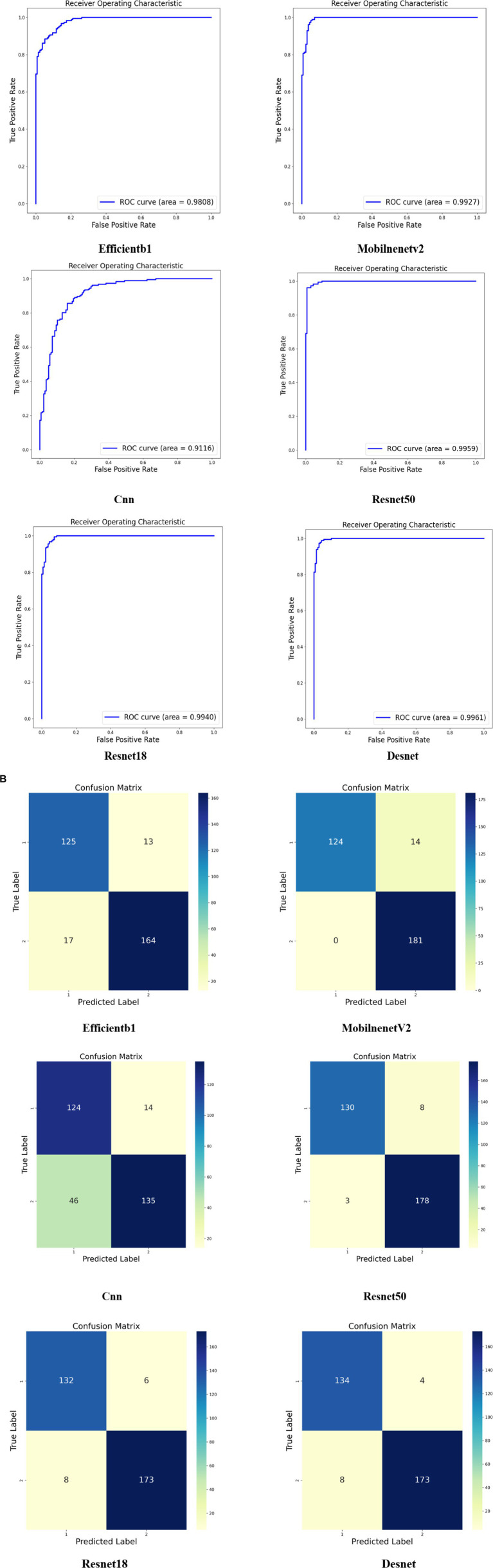
Comparative experimental results of different models. **(A)** Displays the ROC curves of the various models, and **(B)** presents the depiction of the confusion matrices for these models.

Moreover, the outcomes of various models are delineated through ROC curves and confusion matrices. This methodology facilitates a thorough assessment of the performance of each model in classifying monkeypox images, delineating their accuracy, sensitivity, and specificity in categorization. These visual depictions further clarify the comparative advantages and limitations of each model. Contrasted with the results in Figure 3, the superior performance of our proposed model is accentuated. The results of the models are displayed in Figure 4, where Figure 4A illustrates the ROC curve outcomes for the comparative models, and Figure 4B presents the confusion matrix results for the same.

Beyond outperforming other models on major performance metrics, our model is also more user-friendly in operation due to its reduced number of tunable parameters and enhanced computational efficiency. This means that in practical applications, especially when dealing with large datasets, our model can be trained and inferred more rapidly. Furthermore, given its outstanding performance, the potential application spectrum of the model is expanded across various clinical settings, ranging from assisting preliminary diagnoses to providing second opinions, particularly valuable in regions with a scarcity of expert resources.

### 3.3 Ablation experiment result

In our ablation study, detailed experimental validations were conducted for each discrete component, as depicted in Table 2. The table demonstrates a stepwise integration of model components into the baseline. The adoption of the FPN component led to an approximate 1.3% enhancement in model performance, along with a 3% increase in the MCC, robustly validating the efficacy of these components. Further incorporation of the attention mechanism on top of the FPN foundation yielded a continuous 1% uptick in accuracy and improvements across all evaluation metrics. Finally, the implementation of model distillation with vgg16 further elevated the overall performance of the model, thereby confirming the superior performance and robustness of our proposed model.

**Table 2:** Ablation experiment results.

	F1-score	Precision	MCC	Accuracy
Baseline	0.9613	0.9613	0.9106	0.9561
Baseline + FPN	0.9728	0.9572	0.9365	0.9687
Baseline + FPN + mutilattention	0.9805	0.9888	0.9556	0.9781
Baseline + FPN + mutilatt + vgg16	0.9834	0.9834	0.9617	0.9812

**Figure 5:**
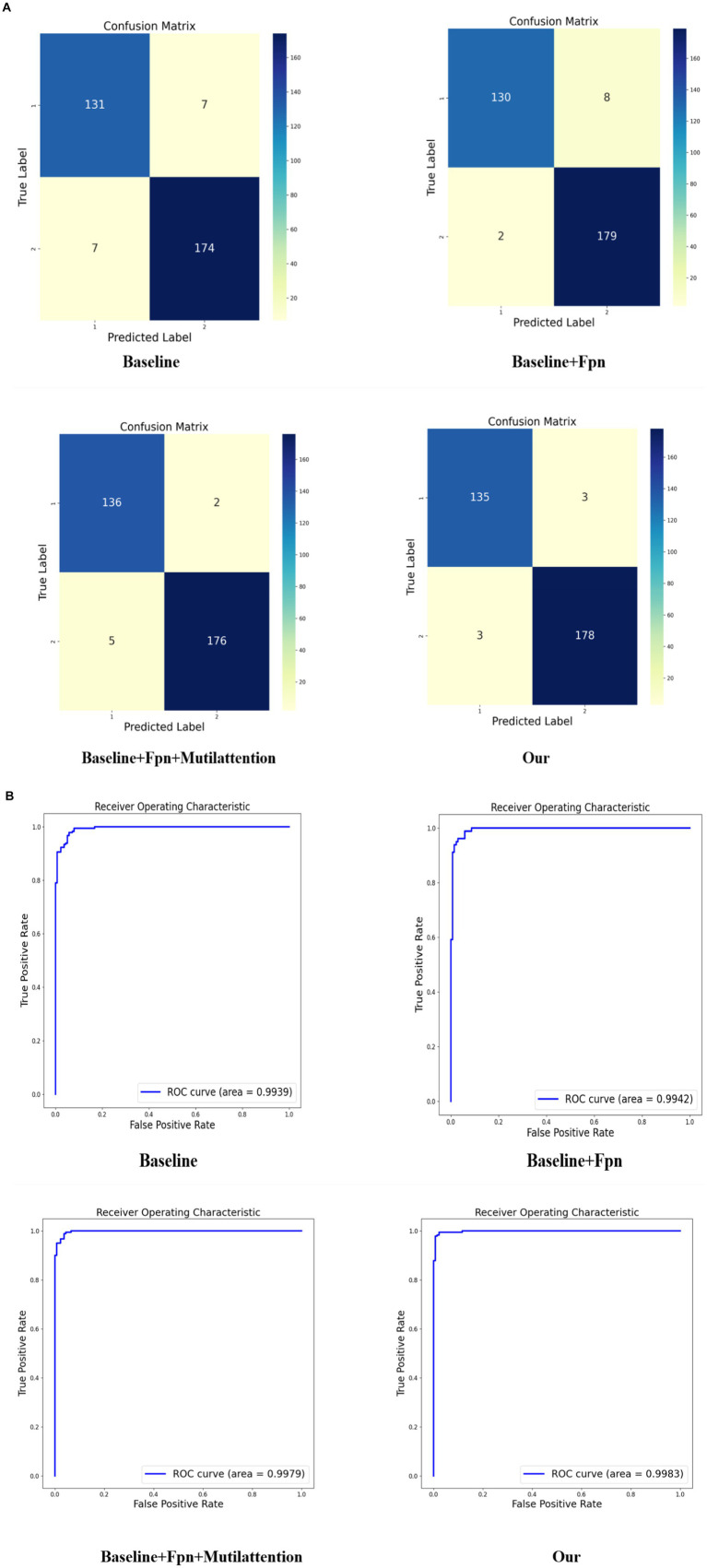
Results of ablation experiment. **(A)** Presents the confusion matrix results of incorporating different components into various models, while **(B)** displays the ROC curve results of incorporating different components into various models.

In Figure 5, we present the confusion matrix results from the ablation study of four different model configurations. The first matrix depicts the baseline model, which correctly predicted class 1 for 131 instances and class 2 for 174 instances, but exhibited 7 misclassifications for each class. The second matrix illustrates the outcomes after incorporating the Feature Pyramid Network (FPN) into the baseline, where we observe a slight decrease in accurate predictions for class 1 but an increase to 179 correct predictions for class 2, highlighting the FPN’s contribution to enhancing classification performance for the latter. The third matrix shows the results upon adding both FPN and multi-head attention mechanisms to the baseline model. This confusion matrix indicates an increase to 136 correct predictions for class 1 and 176 for class 2, demonstrating the further improvements in classification accuracy provided by the multi-head attention mechanism. Finally, the fourth matrix represents our complete model, showcasing the performance after the integration of VGG16, with high accuracy in classifying both classes—135 correct predictions for class 1 and 178 for class 2.

Comparing these four confusion matrices clearly delineates the positive impact of each incremental model enhancement, particularly in reducing misclassifications. Our comprehensive model significantly improves recognition capabilities for class 2 while maintaining high accuracy for class 1, validating the effectiveness and superiority of our proposed model.

In Figure 5B, we present the ROC curve results of the ablation experiment. The results indicate that each addition of components enhances the AUC metric, underscoring the effectiveness of each component within our model.

### 3.4 Visualization of results

Monkeypox clinical skin manifestations typically present as rashes emerging post-febrile phase, initially appearing as red macules, which subsequently evolve into papules, vesicles, pustules, and eventually scabs. These lesions are often circular or oval-shaped and can be distributed on the face, hands, feet, and other body parts. Symptoms of monkeypox may also include lymphadenopathy, headache, myalgia, and fatigue. Similar to smallpox, the evolution of monkeypox skin lesions follows a synchronous progression, indicating that at any given time point, the lesions across the body are in the same stage of development.

**Figure 6:**
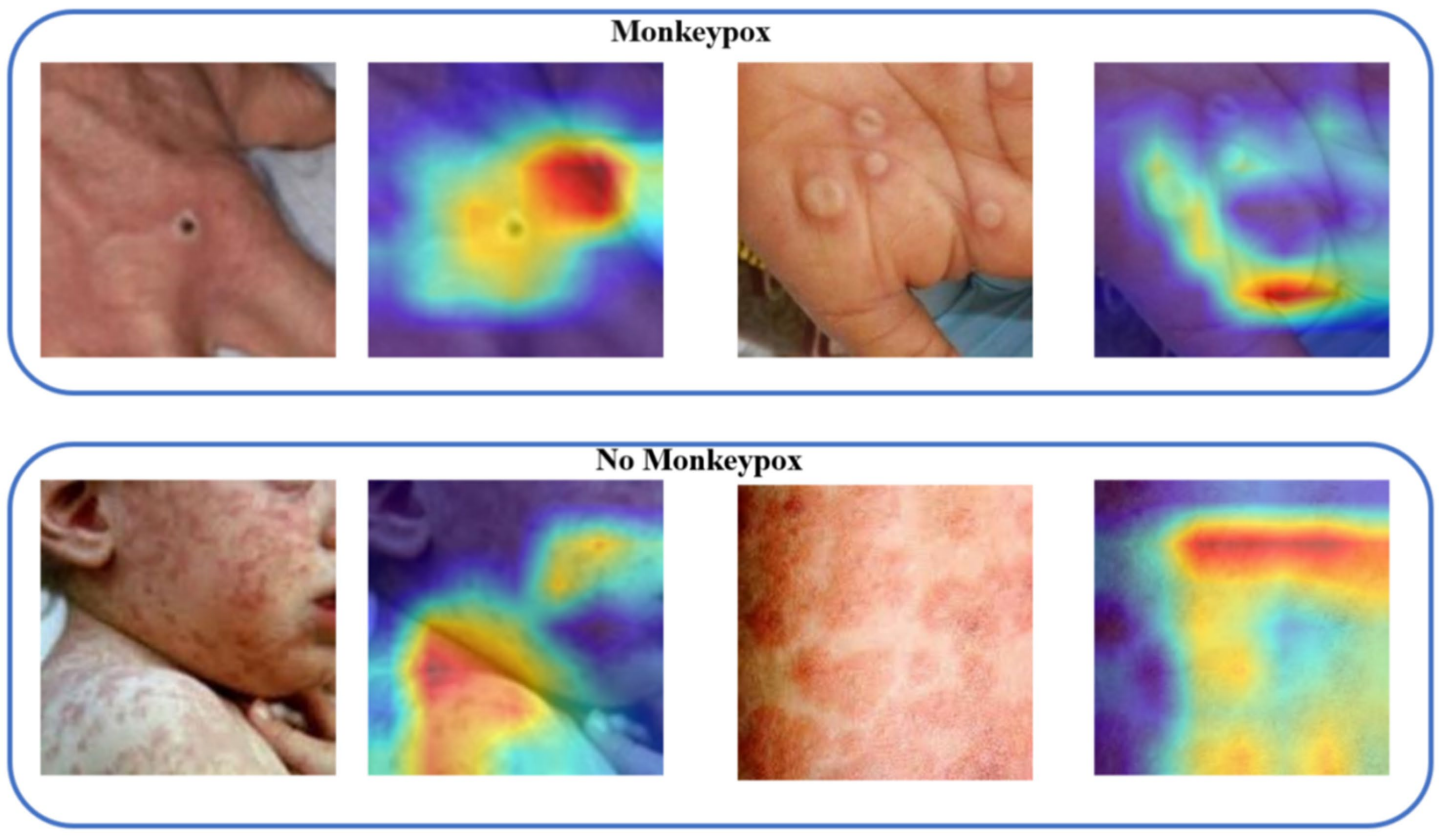
Visualizing the model’s attention regions for monkeypox pathology.

Therefore, we generated attention heatmaps with the objective of visually depicting the areas of focus of the model on monkeypox pathology, as illustrated in Figure 6. These heatmaps serve to highlight the regions within the images that the model prioritizes or deems most relevant in identifying and analyzing the pathological features of monkeypox, providing insight into the model’s decision-making process.

## 4 Discussion

This study implemented a deep learning framework, specifically utilizing a model architecture based on FPN and ResNet18, for the automatic recognition and classification of monkeypox skin lesions. We employed the FPN module to enhance the multi-scale feature extraction capability of the model and augmented this with attention mechanisms to improve feature extraction and visualize the model’s focus areas. Through training and validation, the model demonstrated high accuracy and good generalization capability, particularly in handling diverse images of monkeypox cases. Additionally, we explored model fusion strategies, combining the predictive results of FPNResNet18 and VGG16, to further improve the classification performance.

The outstanding performance of our model in the monkeypox classification task can primarily be attributed to its structural design. The FPNResNet18 architecture enhances the integration of deep and shallow features through pyramid feature maps, effectively capturing the details of the lesion areas. Furthermore, the incorporation of attention modules allows the model to focus more on key areas related to monkeypox pathological features in the images, thereby improving its discriminative capability. From a task perspective, this architecture is particularly well-suited for image classification and pathological feature recognition tasks, as it can effectively handle multi-scale pathological features while maintaining high resolution. Our model demonstrated the best results in both comparative and ablation studies. The comparative experiments validated the superior performance of our model, while the ablation studies confirmed the effectiveness of each component within the model.

From another perspective, this work has the potential to make a significant contribution to the public health sector. By automating the identification and classification of monkeypox cases, the model can help alleviate the workload of medical professionals, accelerating the process of diagnosis and treatment decision-making. Furthermore, the model’s interpretability, facilitated by the visualization of attention mechanisms, provides physicians with additional diagnostic evidence, aiding in the enhancement of diagnostic accuracy and efficiency. In the long term, the development and application of this technology could improve the monitoring and response capabilities for monkeypox disease.

While our work has achieved certain results, it also has limitations. First, the current study focuses solely on the identification of monkeypox types, and future efforts could consider a more detailed classification of different subtypes of monkeypox. Second, our model has not been trained with multi-center data, which may limit its generalizability. Third, as an *in-silico* retrospective image classification task, our study may have inherent biases related to the specific dataset used, which could affect the model’s performance when applied to new data. Finally, the current model has not yet been deployed in practice; future work will need to include further development into a system for clinical translation to facilitate its application in real-world medical settings. Prospective intervention studies will be crucial to validate the model’s effectiveness and address potential biases before practical implementation.

## 5 Conclusion

This study developed a deep learning model that integrates the Feature Pyramid Network (FPN) and ResNet18 architectures, and employed model fusion with the VGG model to automate the identification and classification of monkeypox skin lesions. This integrated approach leverages the multi-scale feature extraction capability of FPN, the deep residual learning of ResNet18, and the robust visual feature recognition of VGG, significantly enhancing the model’s performance. The clinical significance of this research lies in its potential to simplify the diagnostic process, reduce the workload of medical professionals, and improve the accuracy and efficiency of monkeypox diagnosis through automated and enhanced interpretative analysis. Looking forward, the work will further refine the model to differentiate between various subtypes of monkeypox and plans to use multi-center data to enhance its generalizability, moving toward deployment in real clinical settings. The ultimate goal is to integrate this technology into healthcare systems, improving the monitoring and management of monkeypox disease, and strengthening the resilience of public health systems against emerging infectious diseases.

## Data Availability

Publicly available datasets were analyzed in this study. This data can be found at: https://www.kaggle.com/datasets/nafin59/monkeypox-skin-lesion-dataset.
